# The Spirometer

**Published:** 1878-05

**Authors:** 


But it is an instructive fact, that if this easiness of fatigue in ascents, be conjoined with the quick pulse, and be so for months in succession, it is an impressive warning of coming consumption, and millions would be saved, if it were heeded.

A man may be lazy, it may be summer time, or various other things may give rise to a transient exhibition of acceleration in pulse and breath, or they may arise from the mere habit of sedentariness; but there is one easy, decisive, infallable method of determining whether these symptoms are from transient causes, or from an actual change going on in the structure of the lungs themselves; and that is, by measuring the quantity of air which the lungs are capable of drawing in, at one deep, full, free breath, that is done by the use of an instrument often seen in the street, "The Lung Measurer," or "Spirometer," as Mr. Hutchinson, of London, its inventor, names it. The first instrument of the kind ever made in the United States, was made for the Author of these pages, in 1847, since which time a number of eminent English practitioners have learned to employ them, and some few in this country. As a general thing, it has not found favor here, as it is expensive, is liable to abuse, and to the mass of physicians, the opportunities of making varied observations upon it, are not offered. Besides, it requires time and patience to classify the phenomena which it presents; and unless a man have a considerable practice of that kind, it does not pay, either in money or in data for scientific results.

In the Author's practice, then, the great preponderating indications of consumption are, accelerated pulse and breathing; no judicious practitioner will rely wholly on the pulse, or any other two or three symptoms, but on the whole set of symptoms which any given case presents, together with the history of his life, his temperament, his habits, his hereditary tendencies and idiosyncracies, that is, peculiarities, of constitution.

The more obvious symptoms of consumption have been already sketched in a general way. So few persons recover from what is called confirmed consumption, that it was not considered profitable to enter into a critical enunciation and description of all the symptoms, real or imaginary, and in their various stages, degrees and progress; such a thing would materially detract from the practical, utilitarian design, which has been ever prominent. There is so little hope of clearing out the Augean stable of a confirmed consumptive, in any given case, that it is considered only worth while to direct attention critically to the symptoms and stages which admit of a comparatively speedy and permanent arrest or cure.

The large majority of deaths by consumption, are out of married life, indicating the general fact, that its victims are mainly the young, from twenty to thirty. As dying at twenty, or soon after, proves the actual existence of the disease in its forming stages, while yet in the teens, our hope lies in parental influence and intelligence, for then, they can enforce by authority, that course of life, most appropriate towards arresting and removing the disease.

